# Novel type I interferon IL-28A suppresses hepatitis C viral RNA replication

**DOI:** 10.1186/1743-422X-2-80

**Published:** 2005-09-07

**Authors:** Haizhen Zhu, Mike Butera, David R Nelson, Chen Liu

**Affiliations:** 1 Department of Pathology, Immunology and Laboratory Medicine, University of Florida, P. O. Box 100275, Gainesville, Florida 32610, USA; 2 Department of Medicine, University of Florida, P. O. Box 100275, Gainesville, Florida 32610, USA

## Abstract

Interferon alpha (IFN-α)-based therapy is the currently approved treatment for chronic hepatitis C viral infection. The sustained antiviral response rate is approximately 50% for genotype-1 infection. The major challenge to the HCV community is to improve antiviral efficacy and to reduce the side effects typically seen in IFNα-based therapy. One of the strategies is to identify new interferons, which may have better efficacy and less undesirable side effects. In this report, we examined the role of IL-28A (IFN λ2), a novel type I IFN, in suppression of human hepatitis C viral RNA replication. We have cloned both the human genomic DNA and cDNA of IL-28A, and evaluated their biological activity using HCV RNA replicon cell culture system. The results show that IL-28A effectively inhibits HCV subgenomic RNA replication in a dose-dependent manner. Treatment of human hepatoma cells with IL-28A activates the JAK-STAT signaling pathway and induces the expression of some interferon-stimulated genes (ISGs), such as 6–16 and 1–8U. We also demonstrate that IL-28A induces expression of HLA class I antigens in human hepatoma cells. Moreover, IL-28A appears to specifically suppress HCV IRES-mediated translation. Although IL-28A receptor shares one subunit with the IL-10 receptor, IL-10 treatment has no detectable effect on IL-28A-induced antiviral activity. Interestingly, IL-28A can synergistically enhance IFNα antiviral efficacy. Our results suggest that IL-28A antiviral activity is associated with the activation of the JAK-STAT signaling pathway and expression of ISGs. The effectiveness of IL-28A antiviral activity and its synergistic effect on IFN-α indicate that IL-28A may be potentially used to treat HCV chronic infection.

## Background

Interferon alpha (IFN-α), the prototype of type I interferon, is widely used to treat human viral infections and certain malignant tumors [1]. There are several subtypes of type I interferons in humans, namely IFN-α, IFN-β, IFN-ω, IFN-κ, IFN-tau, IFN-epsilon, IFN-zeta, and the recently discovered IFN-λ [2,3]. At least 13 nonallelic IFN-α genes, a single IFN-β gene, and a single IFN-ω gene were identified on human chromosome 9 [4,5]. There are three genes for IFNλ, named as IFN-λ1, IFN-λ2, and IFN-λ3 (also referred to as IL-29, IL-28A, and IL-28B, respectively). Expression of these interferons is induced by viral infection in the majority of nucleated cells. All the type I interferons possess antiviral activity, but the antiviral efficacy appears to vary significantly in subtypes [6,7]. They play a critical role in the innate and adaptive immune responses to viral infection [8]. Interferons exert their biological activities by binding to the heterodimeric receptor. Current evidence suggests that all the type I interferons, except for IFNλ, utilize the same cell membrane-bound receptor, IFNAR, consisting of two subunits, IFNAR1 and IFNAR2. The binding of the receptor by type I interferons predominantly activates The JAK-STAT signaling pathway [9], although other signaling pathways can also be activated in some types of cells [10,11]. Activation of the JAK-STAT pathway leads to induction of the IFN-stimulated gene factor 3 (ISGF), consisting of STAT1, STAT2, and IFN-regulatory factor 9 (IRF-9), which serves as a transcription complex to induce the expression of the downstream target genes, referred to as interferon-stimulated genes (ISG) [12,13]. In either virus-infected or non-infected cells, IFNs induce the transcription of more than 1000 genes [14,15], some of which have been shown to possess direct antiviral properties [16-18]. Moreover, recent studies suggest that type I interferons have an impact on adaptive immunity by regulating MHC class I antigen expression, stimulating dendritic cell maturation [19], and increasing the function of the natural killer (NK) cells [20].

The three members of novel IFNλ have several unique features: 1. The sequence homology of IL-28 and other type I interferons is only 15–19%; 2. These genes contain introns; 3. They bind a specific heterodimeric receptor: one subunit belonging to the class II receptor family and the other subunit is identical to the IL-10 receptor subunit 2; 4. The receptor expression exhibits dramatic variations in different tissues; and, 5. The genes are located on chromosome 19 (q13.13). Despite these unique features of IFN-λ, initial studies have demonstrated that these interferons can be activated by double-stranded RNA and viral infection in cell cultures [2,3]. These interferons suppressed the replication of vesicular stomatitis virus (VSV) and encephalomyocarditis virus (ECMV) in human cell lines, activated the JAK-STAT pathway, and induced expression of some ISGs, which are similar to all the other type I interferons. Thus, it is important to thoroughly investigate these interferons, and to explore the possibility of potential clinical application.

Hepatitis C viral (HCV) infection is a global health problem. It infects more than 170 million people worldwide and 4 million people in the United States [21]. There is no effective vaccine available [22], and the current treatment is the combination therapy with interferon alpha (IFN) and a nucleotide analog, Ribavirin. The best response rate for genotype 1 infection, the predominant viral strain in the United States, is about 50% [23-25]. Moreover, IFN treatment carries significant side effects, partially due to the broad range of IFN biological activities [26]. Unfortunately, the mechanisms of interferon antiviral action, as well as the mechanisms of viral interferon resistance, are still poorly characterized. Thus, a major challenge to the HCV community is to improve therapy for IFN nonresponders, and to reduce its side effects. One of the strategies is to identify new interferons or biological molecules, which may have better efficacy and less undesirable side effects.

In this report, we examined the role of IL-28A in suppression of human HCV RNA replication. We cloned both the human genomic DNA and cDNA of IL-28A, and evaluated their biological activities, cell signaling pathway, and gene induction using HCV RNA replicon cell culture system. We also examined the interactions of IL-28A, IFN, and IL-10.

## Results

### Cloning of IL-28A genomic DNA and cDNA

**Figure 1:**
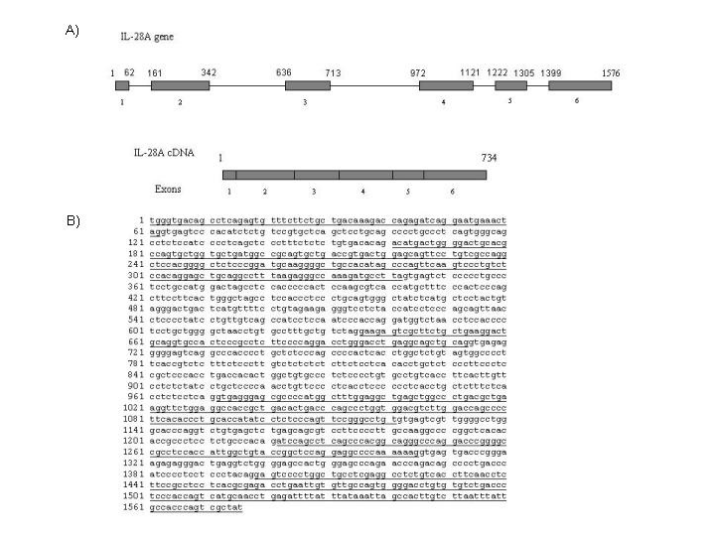
**IL-28A gene structure**. A) Schematics of the exon-intron structure of the gene. The numbers indicate exon location. B) Complete sequence of IL-28A genomic sequence (Accession number DQ126336). The bold nucleotides are the nucleotide sequence of the exons (Accession number DQ126337).

To clone the cDNA of IL-28A, we designed primers according to the available sequence information in Genbank (NM_172138). Total RNA was isolated from normal human splenic tissue, followed by RT-PCR amplification. An intense and specific DNA fragment of 1.6 kb in size was identified, which was larger than the predicted 0.7 kb for IL-28A cDNA. DNA sequence analysis confirmed that this fragment represented the genomic DNA of IL-28A gene (Fig. 1). This 1.6 kb PCR reaction product must be derived from the residual DNA in the total RNA preparation. Consistent with this assumption, extensive DNase I treatment of the RNA preparation eliminated the amplification. Because we could not amplify the cDNA fragment using this approach with several attempts, we, therefore, decided to clone the cDNA fragment using the IL-28A genomic clone. First, the IL-28A genomic DNA was cloned into the expression vector pEF/V5-His-TOPO to construct the plasmid, pTOPO-IL-28A. We then transfected pTOPO-IL-28A into Huh-7 cells, followed by RNA isolation and vigorous DNase I digestion. RT-PCR was performed using the same pair of primers described above. A 0.7 kb cDNA fragment was readily obtained, which corresponded to the predicted cDNA size of IL-28A. We sequenced and analyzed both the genomic fragment and its cDNA. As shown in Fig. 1A, there are five introns and six exons in the IL-28A gene. The first ATG starts from nt.53 and ends at nt.655 (Fig 1B). The predicted amino acid sequence was identical to that published by Sheppard et al. [3].

### IL-28A exhibits anti-HCV activity

**Figure 2:**
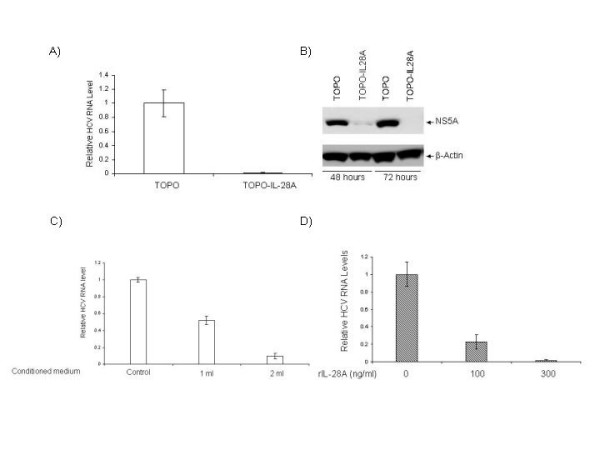
**Effects of IL-28A on HCV RNA replication and protein expression**. A) GSB cells were transfected by either control plasmid (TOPO) or IL-28A genomic expression construct (TOPO-hIL28A). After 48 hours, total RNA was isolated, followed by real-time PCR analysis with HCV-specific primers. The data represents the normalization with the internal control GADPH. B) Western blot analysis of GSB1 cells transfected with the control plasmid (TOPO) or IL-28A expression construct (TOPO-IL28A). The monoclonal antibody is specifically against HCV NS5A. The internal control is actin. C) Effect of IL-28A-conditioned medium on HCV RNA replication in GSB cells. The conditioned medium was used to treat the cells for 48 hours, followed by real-time RT-PCR analysis. D) Effect of recombinant IL-28A on HCV RNA replication in GSB cells. The relative HCV RNA levels were normalized with the internal control GADPH. The error bars indicate the variations of three independent assays.

Since IL-28A is a new member of type I interferon family and IFNα is widely used to treat HCV infection, it is logical to examine its anti-HCV activity. We first tested whether the IL-28A DNA is functional in human hepatoma cells. The eukaryotic expression vectors containing either the genomic DNA or cDNA were transfected into a HCV subgenomic replicon cell line, GSB1. The control cells were transfected by pTOPO vector without any insert sequence. Forty-eight hours after transfection, total RNA was isolated, followed by real-time RT-PCR analysis. As shown in Fig. 2A, compared with the control plasmid-transfected cells, the IL-28A transfected cells had significantly lower viral RNA copy numbers. The viral suppression effect was also demonstrated by viral NS5A protein expression, as determined by Western blot analysis (Fig. 2B). To further determine the effect of IL-28A secreted by cells, we then tested the antiviral effect of the conditioned medium. We subcloned IL-28A cDNA into pEF/V5-His-TOPO vector and generated the plasmid, pTOPO-IL-28A07. The plasmid pTOPO-IL-28A07 was transfected into Huh-7 cells, and the supernatant was harvested after 72 hours of incubation. Varying amounts of the conditioned-medium from Huh7 cells transfected with plasmid pTOPO or pTOPO-IL-28A07 were added to GSB1 cells. After 48 hours of incubation, total RNA was isolated from the cells, followed by real-time RT-PCR analysis. As shown in Fig 2C, the IL-28A-conditioned medium demonstrated a significant inhibitory effect on HCV RNA replication in a dose-dependent manner. Similar results were also obtained using pTOPO-IL28A, the genomic expression construct (data not shown). We then further examined the effect of the recombinant IL-28A on HCV RNA replication by incubating GSB1 cells with varying doses of rhIL-28A, followed by total RNA extraction and real-time PCR analysis. As shown in Fig. 2D, the replication levels of HCV RNA were significantly suppressed by rhIL-28A. Again, IL-28A inhibits the viral RNA replication in a dose-dependent manner, but the effective dose of rhIL28A is significantly higher than recombinant IFN.

**Figure 3:**
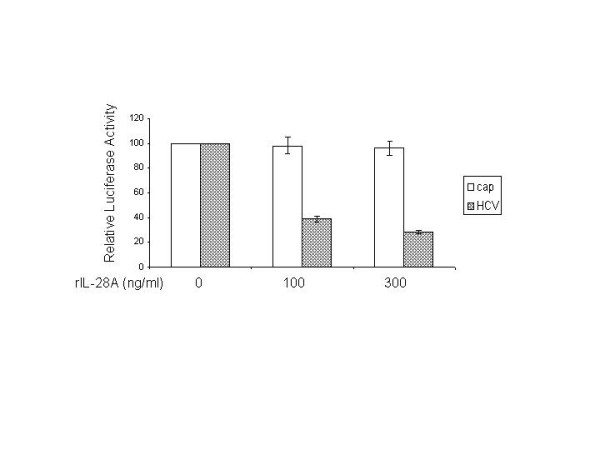
**Effects of IL-28A on CAP-dependent and HCV IRES-dependent translation**. The GSB1 cells was transfected with control plasmid or plasmid pRL-HL, which has different luciferases directed by either CAP- or HCV IRES. After 48 hours of transfection, the cells were treated with varying doses of IL-28A. Cell extracts were made after 24 hours of incubation, followed by luciferase determination. The data represents the average of three independent experiments. The open column indicates CAP-mediated translation. The shadowed column indicates HCV IRES-mediated translation. The error bars indicate the variations of three independent assays.

For simultaneous assessment of cap-dependent and HCV IRES-dependent translation, Huh7 cells were transiently transfected with a bicistronic reporter plasmid, pRL-HL, encoding the Renilla and firefly luciferase cDNAs translated from the 5'cap and internally from the HCV IRES, respectively, for 24 hours, followed by 24 hours of incubation in medium alone or with medium containing increasing amount of hrIL-28A. Cells were harvested, and protein extracts were prepared, and a dual luciferase assay using the luciferase assay system was performed. As shown in Fig. 3, translation from the viral IRES elements exhibited a dose-dependent suppression, while the cap-dependent translation is not significantly affected by IL-28A. These data suggest that IL-28A appears to specifically inhibit HCV IRES-mediated translation without affecting cap-mediated translation in the host cells.

### IL-28A activates the JAK-STAT signaling pathway

**Figure 4:**
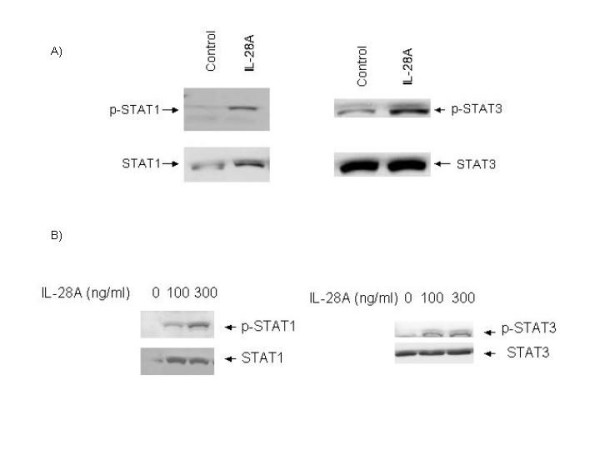
**The effect of IL-28A on JAK-STAT signaling pathway**. GSB1 cells were treated with either IL-28A conditioned medium (A) or recombinant rIL-28A (B) for 30 minutes, followed by protein extraction and Western blot analysis using antibodies as indicated in the figure. Equal amounts (20 ug) of proteins were loaded in each lane and confirmed by detection of actin. STAT1 or STAT3 indicates total STAT protein. p-STAT1 or p-STAT3 indicates tyrosine phosphorylated form (activated STAT protein). The figures are representatives of at least four independent experiments.

It is known that type I interferons initiate cellular responses at least partially through the JAK-STAT pathway. All the human type I IFNs interact with the same receptor, IFNAR [27]. When IFNs bind to specific cell surface receptors on the host cells, the IFNAR receptor complex will activate the JAK proteins, JAK1 and TYK2. The activated-JAK proteins then phosphorylate STAT1, STAT2, and STAT3. We hypothesized that the IL-28A-induced antiviral effect in GSB1 cells would depend upon the activation of the JAK-STAT signaling pathway. We, therefore, analyzed the status of STAT1 and STAT3 in response to IL-28A stimulation. Huh7 or GSB1 cells were treated with pTOPO-IL-28A07 conditioned medium or rhIL-28A for 30 minutes, followed by total protein extraction and Western blot analysis using anti-p-STAT1, anti-p-STAT3, total STAT1 and STAT3 monoclonal antibodies. As shown in Fig. 4, both phosphorylated STAT1 and STAT3 were detected in cells treated with IL-28A. This result indicates that IL-28A utilizes the similar JAK-STAT signaling pathway as the IFN-α and IFN-β, despite receptor differences.

### IL-28A induces interferon stimulated genes (ISGs) expression

**Figure 5:**
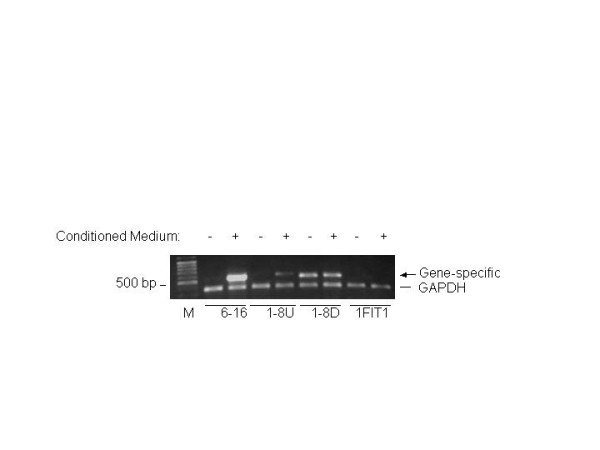
**Induction of interferon stimulated genes by IL-28A in GSB1 cells**. GSB1 cells were treated by either control or 2-ml IL-28A-conditioned medium for 12 hours. Total RNA was isolated, followed by RT-PCR analysis using a pair of gene-specific primers and a pair of DADPH primers. The PCR amplification cycle is 25, which ensures PCR reaction in linear range. The PCR products were analyzed in 1% agarose gel. M indicates the DNA molecular weight marker. The arrow indicates gene-specific products. The bar indicates GADPH DNA fragment. The figure is a representative of two independent assays.

The transcription factor IFN-stimulated gene factor 3 complex (ISGF3), consisting of phosphorylated STAT1, phosphorylated STAT2, and IRF-9/p48, translocates into the nucleus and binds to IFN-stimulated response elements (ISRE) within the promoters of ISGs [9]. Interferons exert their biological function through induction of ISGs in the cell. Therefore, it is possible that IL-28A provides antiviral activity by induction of a subset of IFN-stimulated genes (ISGs). To determine whether IL-28A can induce the ISGs, total RNA was isolated from the cells treated by IL-28A-conditioned medium from Huh7 cells transfected with pTOPO-IL-28A, followed by semi-quantitative RT-PCR analysis using gene specific primer sets for 6–16, 1–8U, 1–8D, and IFIT1. As shown in Fig. 5, 6–16 and 1–8U were significantly induced by IL-28A, while the gene IFIT1 was not effectively induced. This observation suggests that IL-28A is capable of inducing ISGs, but the gene profile may not be identical to that of IFN.

### IL-10 has no effect on the IL-28A-induced anti-HCV activity

**Figure 6:**
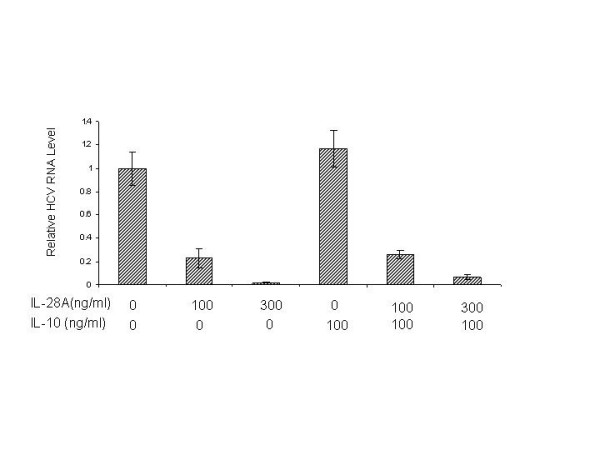
**Effect of IL-10 on IL-28A-induced antiviral activity**. The GSB1 cells were treated with varying doses of IL-10 or IL-28A as indicated for 72 hours. Total RNA was isolated for real-time PCR analysis using HCV-specific primers. The data represents the normalization with internal control GADPH. The error bars indicate the variations of three independent assays.

The IL-28A receptor complex consists of a ligand-binding chain, IL-28R, and an accessory receptor chain, IL-10R2. So it is logical to determine whether IL-10 interferes with IL-28A in inhibiting HCV RNA replication. GSB1 cells were treated with or without IL-28A (100 ng/mL and 300 ng/mL) in the presence or absence of 100 ng/mL IL-10. After 72 hours of incubation, total RNA was isolated from the cells, followed by real-time RT-PCR analysis. As shown in Fig. 6, IL-10 did not have a significant effect on the IL-28A-induced anti-HCV activity, while IL-28A can decrease RNA replication,

### IL-28A synergies with IFN-α in suppressing HCV RNA replication

**Figure 7:**
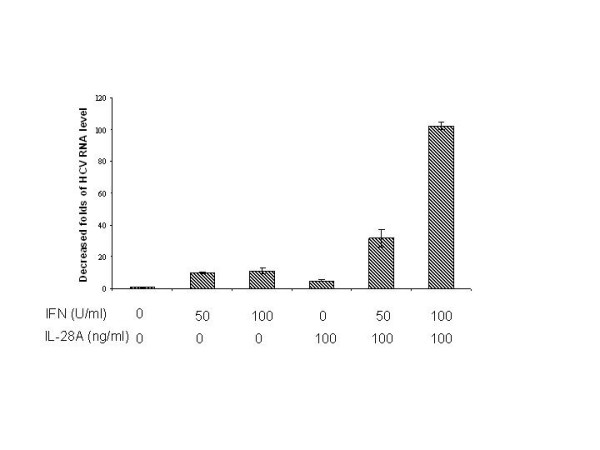
**Effect of IL-28A on the antiviral activity of IFNα**. Varying doses of IL-28A and IFNα2b, either alone or in combination, were added to the GSB1 cells and incubated for 48 hours. Total RNA was isolated for real-time PCR analysis. The vertical axis represents the fold of viral RNA reduction by IL-28A or IFN. The data represents the results of normalization with the internal control GADPH.

**Figure 8:**
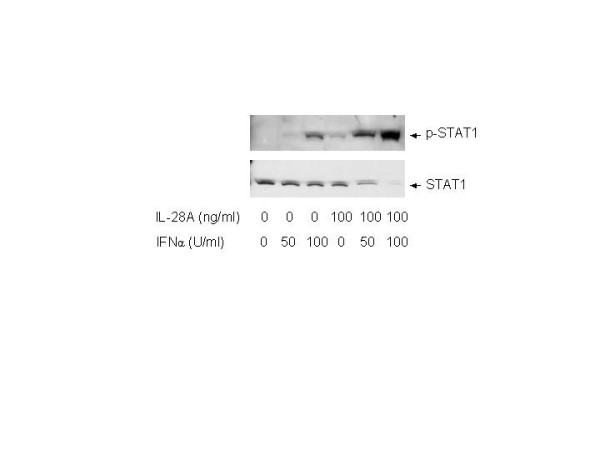
**Effect of IL-28A on IFNα-induced STAT1 activation in GSB1 cells**. GSB1 cells were treated with IL-28A or/and IFN as indicated. After 30 minutes of incubation, total protein was extracted for Western blot analysis using antibodies against total STAT1 (STAT1) or phosphorylation-specific STAT1 (p-STAT1).

The above results indicated that IL-28A can signal through the JAK-STAT pathway in a similar manner as to IFN-α. To determine whether IL-28A enhances IFN-α-induced anti-HCV RNA replication, we tested the effect of IL-28A on IFN-α-induced anti-HCV RNA activity using GSB1 cell. IFN-α was used at the dose of 50 U/mL, 100 U/mL with or without 100 ng/mL IL-28A. After 24 hours of incubation, cells were harvested and total RNA was isolated, followed by real-time RT-PCR analysis using HCV-specific primers. As shown in Fig. 7, the combination of IL-28A and IFN-α reduced HCV viral RNA by 100-fold, while IFNα alone reduced the virus by 15-fold and IL-28A alone by 6-fold. Activation of STAT1 protein by phosphorylation is a critical step for IFN-α signaling pathway. In the next experiment, we examined the effect of IL-28A on the IFN-α-induced STAT1 phosphorylation by Western blotting. As shown in Fig. 8, the levels of p-STAT1 were significantly higher than those induced by IFN-α or IL-28A alone. In addition, STAT1 remained phosphorylated for 8 h after stimulation with IFN-α plus IL-28A, while STAT1 phosphorylation induced by IFN treatment alone decreased to undetectable level (data not shown). The results indicate that IL-28A can synergize with IFN-α in suppressing the HCV RNA replication and inducing intracellular antiviral signaling pathway.

### IL-28A induces HLA class I antigen expression

**Figure 9:**
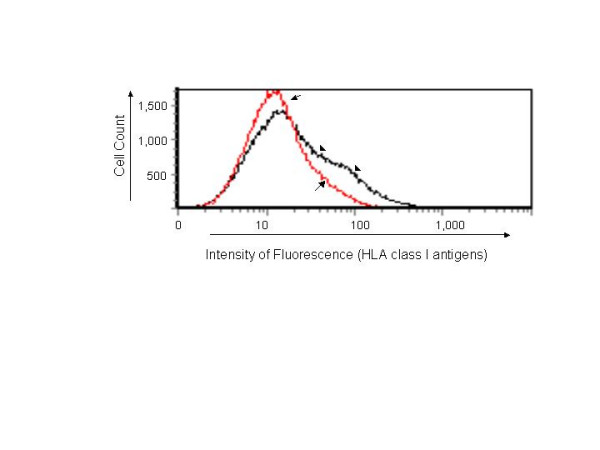
**Effect of IL-28A on HLA class I antigen expression in Huh7 cells**. The Huh7 cells were treated with 2 ml IL-28A-conditioned culture medium for 72 hours. The cells were then harvested and incubated with HLA class I antigen-specific antibody labeled by FITC fluorescence, followed by flow cytometric analysis. The arrow-marked curve indicates control cells. The arrowhead-marked curve indicates cells treated with IL-28A.

Type I interferons are believed to play a role in immune regulation. One of the mechanisms is through induction of HLA class I antigen. To test whether IL-28A has such an effect, we treated Huh7 cells with IL-28A-conditioned medium from Huh7 cells transfected by plasmid pTOPO-IL-28A, followed by flow cytometric analysis using anti-HLA class I antigen. As shown in Fig. 9, treatment with IL-28A induced HLA class I antigen production. The data suggest that IL-28A has a similar capacity to induce class I antigen production as other type I IFN.

## Discussion

Type I interferons play an essential role in innate immune responses against viral infections. There are many subtypes of type I interferons in humans, including the recently identified IFN-λ, consisting of three members, λ1 (IL-29), λ2 (IL-28A), and λ3 (IL-28B). The most extensively studied subtypes are IFN-α and IFN-β. There is relatively little information available for IFN-λ. The major difference between IFN-λ and the other type I IFNs is the utilization of different receptors. Current type I interferon therapy has significant side effects. Identification of novel type I interferons with desirable clinical efficacy and less side effects is needed. IFNλ s are potentially such candidates.

In this report, we have cloned both the cDNA and the gene of human IL-28A. Through a series experiments, we have shown the biological effects of this protein on HCV viral replication, its signaling events in human liver cells, and its interaction with IFN-α and IL-10.

To clone this gene, we employed a RT-PCR approach using total RNA extracted from spleen, liver, and peripheral blood mononuclear cells (PBMC). With extensive effort, we could only obtain IL-28A genomic clones but not cDNA, while we could readily amplify IFN-α and IFN-β cDNA from the same RNA source. We confirmed that the amplified genomic clones were derived from the residual DNA in the RNA preparation, since two rounds of DNase I treatment eliminated the amplification. This indicates that there is no detectable IL-28A expression in these tissues at a normal physiological condition, although it has been reported that IL-28A is expressed in PBMCs from HCV-infected patients [28]. To obtain the cDNA clone, we decided to clone the genomic DNA into a expression vector, and then transfected it into Huh7 cells. Total RNA was extracted from the transfected cells and RT-PCR was performed. The cDNA DNA fragment was easily amplified using this approach. We noticed that Kotenko et al. used a similar strategy to clone the first IL-28A cDNA [2]. By comparing the cDNA and its gene, we identified five introns and six extrons. So far, this is the only type I interferon gene containing introns, while the other type I IFNs encode within a single extron. The presence of multiple introns makes this gene more similar to IL-10 gene family. Interestingly, the IL-28A receptor shares one subunit with IL10 (IL-10Rβ). We know that IL-10 and type I interferons play a different role during the host immune responses to viral infections. The presence of introns generally subjects the gene to an additional gene expression control. According to Kotenco et al., the IL-28A is predominantly expressed in the heart, liver and spleen [2]. Whether the introns play any role in such relatively tissue-restricted expression remains to be investigated.

After cloning this gene, we then showed that the gene product, IL-28A, has similar biological properties as other type I interferons. IL-28A resembles type I IFNs in its ability to induce anti-HCV activity through JAK-STAT signaling pathway. As we have shown in Fig. 4, IL-28A activates both STAT1 and STAT3. The IL-28A-mediated antiviral activity is dose-dependent. Both the recombinant and the gene product produced in liver cells are effective, though the effective dose of the recombinant IL-28A is much higher than the other type I interferons. Similar results were recently reported by other laboratories [29,30].

We further analyzed the expression of ISGs using a RT-PCR approach. Interestingly, at least one ISG cannot be induced by IL-28A, while it can be readily induced by IFNα. Moreover, by testing the effect of interferons on cap-mediated translation and HCV IRES-mediated translation, our preliminary data showed that IL-28A appears to have a selective activity to inhibit HCV-IRES-mediated translation, while it did not affect cap-mediated translation. This observation is consistent with the fact: even at higher dose (1000 ng/ml), IL-28A did not exhibit antiproliferation activity in a human hepatoma cell line (data not shown). These data suggest that IL-28A seems to have at least some different biological activities as compared with IFN-α. Whether these differences can be employed to achieve therapeutic advantage remains to be determined.

As we have mentioned above, the receptor for IL-28A shares a common subunit with IL-10. Our previous study showed that IL-10 did not have direct antiviral activity in patients with chronic HCV infection [31]. We asked the question whether the sharing of a receptor has any impact on IL-28A activity. Our data suggests that IL-10 does not have an antiviral effect in HCV replicon cells, nor does it have any interference with IL-28A antiviral effect. Thus, the significance of receptor sharing remains unknown.

Since IL-28A and other type I interferon use different receptor for signaling transduction, we next examined the combination effect of IL-28A and IFN-α. Interestingly, combination of IL-28A and IFN-α exhibited synergistic effect on JAK-STAT activation and anti-HCV activity. As shown in Figure 7, combination of 50 U IFN-α and 100 ng per milliliter IL-28A reduced HCV RNA by 40 folds, while individual IFN-α and IL-28A reduced HCV RNA by 10-fold and 6-fold, respectively. We do not know the precise mechanism of this synergistic effect, though the STAT1 activation shows the similar synergistic effect (Fig. 8). It is possible that the activation of one receptor may have beneficial effect on the other receptor-mediated pathway. It is also possible that the common downstream molecules shared by both pathways can synergistically induced by these two interferons. This synergistic effect has a significant clinical implication. It is tempting to speculate that combination of these two reagents may have therapeutic benefit for HCV therapy, particularly in the setting of IFN resistance.

Type I interferons have an immunoregulatory function [32,33]. One of the mechanisms is through induction of HLA class I antigens [34]. We tested whether IL-28A has a similar activity. Human hepatoma cells have relatively lower HLA class I antigen expression comparing with normal hepatocytes [35]. Treatment of the hepatoma cells increased class I antigen expression through flow cytometric study. Not only this shows that the IL-28A has immunoregulator effect, but the fact that IL-28A can induce HLA class I antigen in tumor cells may implicate the role of IL-28A in tumor immune therapy. It would be interesting to see whether IL-28A is capable of promoting the host antitumor immunity.

## Conclusion

Our study shows the gene structure of IL-28A, its antiviral effect on HCV, its signaling transduction pathway, and the induction of ISGs. More importantly, we demonstrate the synergistic effect of IL-28A and IFNα on anti-HCV activity, which has a potential clinical application. IFN-α is currently used for the treatment for chronic HCV infection, HBV infection, and many malignant tumors, including hepatitis B, melanoma, hairy cell leukemia, and non-Hodgkin's lymphoma. IL-28A is a potential therapeutic agent to treat these clinical diseases.

## Methods

### Cell cultures, reagents and plasmids

The HCV subgenomic replicon cell line, GSB1, was a gift from Dr. Christopher Seeger [36,37]. All cells were propagated in DMEM supplemented with 10% FBS, 200 μM L-glutamine, nonessential amino acids, penicillin and streptomycin. Culture of the replicon cells has been previously described [15]. The expression vector, pEF6/V5-His-TOPO, was obtained from Invitrogen (Carlsbad, CA). The HCV-NS5A-specific monoclonal antibody was generated in the laboratory. Monoclonal antibodies against actin, STAT1, STAT3 and phosphorylated STAT3 were obtained from Santa Cruz Biotechnology (Santa Cruz, CA). The antibodies against phosphorylated STAT1 were obtained from Upstate (Charlottesville, VA). The secondary antibody goat anti-mouse or anti-rabbit IgG-HRP was from Santa Cruz Biotechnology. Supersignal West Pico Chemiluminescent Substrate was purchased from Pierce Biotechnology, Inc. (Rockford, IL). Recombinant human IL-28A (rhIL-28A) and hIL-10 were purchased from R&D Systems (Mineanapolis, MN). The plasmid pRL-HL (a gift from Dr. Lemon) is a bicistronic expression construct encoding Renilla and firefly luciferase cDNAs translated from 5'cap and internally from the HCV IRES (internal ribosome entry site), respectively [38].

### Amplification of human IL-28A DNA, cDNA, and plasmid construction

RNA was isolated from human spleen. The human IL-28A cDNA was amplified by RT-PCR from human spleen RNA using two primers: 5'-GGGTGACAGCCTCAGAGTG-3', 5'-ATAGCGACTGGGTGGCAATA-3'. Superscript One-Step RT-PCR kit with platinum Taq according to the instructions (Invitrogen). The One-Step RT-PCR conditions were as follows: 50°C, 30 min; 94°C, 4 min; followed with 40 cycles (95°C, 30 s; 55°C, 30 s; 72°C, 1 min;). The IL-28A DNA was ligated into pEF6/V5-His-TOPO vector. The expression vector pTOPO-IL-28A were transfected into Huh7 cells using Lipofectin Reagent (Invitrogen) according to the manufacturer's instruction. The total RNA was purified from Huh7 cells transfected by pTOPO-IL-28A for 48 hours and treated by DNase I. The human IL-28A cDNA was generated by RT-PCR from the total RNA pretreated by DNase using the above primers. The reactions were performed using 72°C, 7 mins. The expression vector pTOPO-hIL-28A 0.7 was constructed by inserting the human IL-28A cDNA into pEF6/V5-His-TOPO.

### Human IL-28A DNA Sequencing

The IL-28A DNA was amplified as described above. The expression vector TOPO-hIL-28A was sequenced using The BigDye Terminator V3.1 Kit from Applied Biosystems (Foster City, CA). The reaction condition was: 96°C, 10 s; 50°C, 5 s; 60°C, 4 min, total 25 cycles. After that, 1/20 volume of 3 M sodium acetate (pH5.2) and 3 times volume of ethanol were added, and incubated at -20°C for 30 mins, followed by spinning down at 13000 g at 4°C for 30 mins. The DNA pellet was washed using 70% ethanol and dried by vacuum. The sequence was detected by ABI PRISM 377 DNA Sequencer (Applied Biosystems).

### DNA transfection

The transfection protocol has been described previously [39,40]. Briefly, GSB or Huh7 cells were transfected with control plasmid pTOPO, pTOPO-IL-28A or pTOPO-IL-28A07 plasmid using Lipofectin. In a 6-well tissue culture plate, 1 × 10^5 ^GSB or Huh7 cells were seeded in 2 ml of DMEM supplemented with serum and incubate at 37°C in an incubator overnight. For each transfection, 2 μg of DNA was used. The plasmid, pTOPO, pTOPO-IL-28A, or pTOPO-IL-28A07 was transiently transfected into GSB or Huh7 cells. The transfected cells were incubated for another 48 hours before experiments.

### Reverse Transcription and Polymerase Chain Reaction (RT-PCR)

Total cellular RNA was purified from cells. After reverse transcription, cDNA was used for PCR. The primers are for 6–16 (G1P3), forward 5'-AACCGTTTACTCGCTGCTGT-3, reverse 5'-GCTGCTGGCTACTCCTCA-3'; for 1–8U, forward 5'-CAAATGCCAGGAAAAGGAA-3', reverse 5'-ATACAGGTCATGGGCAGAGC; for 1–8D, forward 5'-TGCCAGGAA GAGGAAACTGT-3', reverse 5'-CCTCAATGATGCCTCCTGAT-3'; for IFIT1, forward 5'-TCTCAGAGGAGCCTGGCTAA-3', reverse 5'-AGTGGCTGATATCT GGGTGC-3'; for GAPDH, forward 5-TCACCAGGGCTGCTTTTA-3', reverse 5'-TTCACACCCATGACGAACA-3'. The PCR conditions were as follows: 94°C, 4 min; (95°C, 30 s; 55°C, 30 s; 72°C, 1 min;) × 40 cycles; 72°C, 7 mins. The PCR product was detected on 2% agarose gel.

### Quantitative Real-Time PCR

Total cellular RNA was isolated from cells as described before. Real-time PCR was preformed as described previously [39]. Briefly, first-strand cDNAs were synthesized from total cellular RNA by reverse transcription (20 μl of reaction volume) using the Superscript II (50 U reverse transcriptase per reaction) first-strand synthesis for RT-PCR kit (Invitrogen) primed with oligo (dT)_12–18 _(Invitrogen) according to the manufacturer's instructions. Fluorophore-labeled LUX primers and their unlabeled counterparts were obtained from Invitrogen. Reactions were conducted in a 96-well spectrofluorometric thermal cycler (ABI PRISM 7700 Sequence detector system, Applied Biosystems). Fluorescence was monitored during every PCR cycle at the annealing step. The primers for HCV are: 5'-CGCTCAATGCCTGGAGATTTG-3', 5'-GCACTCGCAAGCACCCTATC-3'; for GADPH: 5'-TGCTGGCGCTGAGTACGTC-3', 5'-GTGCAGGAGGCATTGCTGA-3'. PCR conditions were as follows: 50°C, 2 min; 95°C, 10 min; (95°C, 15 s; 60°C, 1 min) × 40 cycles. Results were analyzed with SDS 2.0 software from Applied Biosystems. Results for all experiments represent triplicate determinations. Results are represented as means ± SD.

### Western Blot Analysis

Equal numbers of cells were washed with PBS and lysed in RIPA buffer as described previously [15]. Protein extraction from cells, electrophoresis and Western blot analysis were described previously. Approximately 20 μg of protein were electrophoresed on a 8% SDS-polyacrylamide gel and transferred to polyvinylidene difluoride membrane (Bio-Rad). The membrane was incubated overnight at 4°C in a block buffer (TBS containing 0.1% Tween 20 and 5% fat-free milk power). The blots were probed with monoclonal antibodies specific for NS5A, STAT1, and STAT3, p-STAT3, actin or polyclonal antibody specific for p-STAT1 for 1 hour at room temperature. After being washed 3 times for 30 min each with 0.1% Tween 20 in TBS, the membrane was incubated with the secondary antibody diluted in 5% fat-free milk in TBS containing 0.1% Tween 20 for 1 hour at room temperature and washed 3 times as described above. Proteins were visualized by using Supersignal West Pico Chemiluminescent Substrate.

### Flow cytometry

To detect the expression of MHC class I antigen, Huh7 cells were treated with IL-28A conditioned medium from Huh7 cells transfected by plasmid pTOPO-IL-28A for 72 hours and their MHC class I expression was analyzed by flow cytometry as previously described [41]. Cell surface expression of the HLA class I antigens were detected using class I antibody, followed by fluorescein isothiocyanate (FITC)-conjugated goat anti-rabbit IgG. Ligand binding was detected by flow cytometry.
